# Supplementary figures and images for: F4-Neuroprostane Effects on Human Sperm
Source: Int J Mol Sci. 2023 Jan 4;24(2):935. doi: 10.3390/ijms24020935 (PMC9861396; doi:10.3390/ijms24020935)

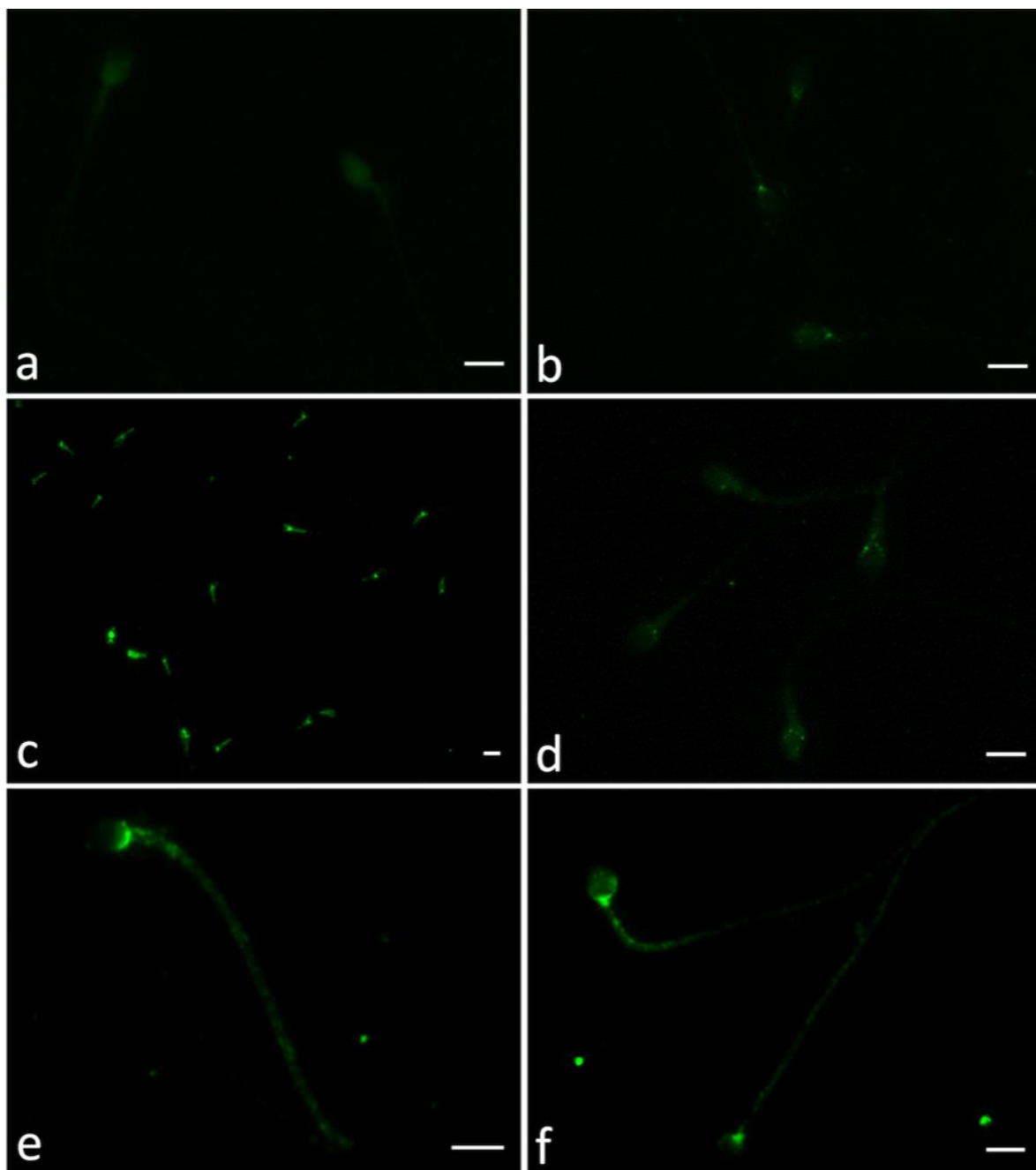

Supplement: Supplementary file 1 [file ijms-24-00935-s001.zip › ijms-2046884-supplementary.pdf]
